# First experimental evidence of the piezoelectric nature of struvite

**DOI:** 10.1038/s41598-021-94410-2

**Published:** 2021-07-21

**Authors:** Jolanta Prywer, Rafał Kruszyński, Marcin Świątkowski, Andrzej Soszyński, Dariusz Kajewski, Krystian Roleder

**Affiliations:** 1 Institute of Physics, Lodz University of Technology, ul. Wólczańska 219, 90-924 Łódź, Poland; 2 Institute of General and Ecological Chemistry, Lodz University of Technology, Żeromskiego 116, 90-924 Łódź, Poland; 3 Institute of Physics, University of Silesia, 75 Pułku Piechoty 1, 41-500 Chorzów, Poland

**Keywords:** Materials science, Condensed-matter physics

## Abstract

In this paper, we present the first experimental evidence of the piezoelectric nature of struvite (MgNH_4_PO_4_·6H_2_O). Using a single diffusion gel growth technique, we have grown struvite crystals in the form of plane parallel plates. For struvite crystals of this shape, we measured the piezoelectric coefficients *d*_33_ and *d*_32_. We have found that at room temperature the value of piezoelectric coefficient *d*_33_ is 3.5 pm/V, while that of *d*_32_ is 4.7 pm/V. These values are comparable with the values for other minerals. Struvite shows stable piezoelectric properties up to the temperature slightly above 350 K, for the heating rate of 0.4 K/min. For this heating rate, and above this temperature, the thermal decomposition of struvite begins, which, consequently, leads to its transformation into dittmarite with the same non-centrosymmetric symmetry as in case of struvite. The struvite-dittmarite transformation temperature is dependent on the heating rate. The higher the heating rate, the higher the temperature of this transformation. We have also shown that dittmarite, like struvite exhibits piezoelectric properties.

## Introduction

Struvite is a mineral first identified and described by Ulex in 1845^1^. Struvite is magnesium ammonium phosphate hexahydrate, the chemical formula of which is MgNH_4_PO_4_⋅6H_2_O. It crystallizes in the orthorhombic system, in space group P*mn2*_1_ and has the following unit cell parameters: *a* = 6.9650(2) Å, *b* = 6.1165(2) Å, *c* = 11.2056(3) Å^2^. Struvite forms transparent crystals that can become white, which is a sign of a gradual loss of water of crystallization.

In the light of extensive research, it can be stated that struvite has been studied for several reasons. First of all, struvite is the most common mineral found in infectious urinary stones. Infectious urinary stones are the result of urinary tract infection with urease-producing bacteria, mainly of the *Proteus* species. Urease is a bacterial enzyme that breaks down urea, physiologically present in urine, into carbon dioxide and ammonia. This decomposition initiates a whole series of chemical reactions that lead to the crystallization of various solid phases, of which struvite is the main phase^3–6^. Such stones pose a serious problem, the more so as a continuous increase in the incidence of infectious stones has been observed, especially in highly developed countries.

Struvite also poses a problem in wastewater treatment plants because it easily precipitates in specific places, clogging pipes, and other devices^7–13^. Struvite formation is strongly related to the wastewater pH level; the higher the pH, the greater the crystallization potential of struvite. On the other hand, struvite is a potential source of phosphorus, nitrogen, and magnesium, therefore it is the main compound recovered from wastewater and transformed into a useful fertilizer containing these elements (e.g. Refs.^14–17^). Struvite recovery from wastewater is particularly important due to phosphorus^10,18,19^ because natural sources of phosphorus free from heavy metals are ever more rare. Phosphorus, nitrogen, and potassium are the three main elements necessary for plant life. That is why many fertilizers contain phosphates, and phosphorus recovery from wastewater, in particular from struvite, proves very important.

Recently, struvite has been found to be a ferroelectric^20^. Ferroelectric properties, in particular spontaneous polarization, as indicated in Ref.^20^, may affect the interaction of bacteria with struvite crystals in urine, as well as interaction with admixtures inhibiting or accelerating the growth of these crystals. Struvite, as a ferroelectric crystal, should also be a piezoelectric crystal. Piezoelectrics are materials in which an electric field can be generated by mechanical stress applied. Piezoelectrics also exhibit reverse piezoelectric phenomenon, consisting of the appearance of mechanical stress (change in crystal size) under the influence of an applied electric field. The piezoelectric effect was discovered in 1880 by brothers Pierre and Jacques Curie^21^. There are different types of materials that exhibit piezoelectric properties. Piezoelectric materials can be in the form of single crystals (the best known natural piezoelectric material is quartz, SiO_2_), polycrystals (usually ceramics), and non-crystalline materials (such as polymers)^22^. There are different mechanisms causing the piezoelectric effect. Crystalline materials exhibit a piezoelectric effect due to their unique crystal structure. Struvite is a single crystal and additionally a mineral and belongs to the *mm*2 point group, which is one of the 20 point groups in which the piezoelectric effect is possible.

The aim of the research presented in this paper is the experimental measurement of the piezoelectric effect of struvite depending on temperature, and its description. This phenomenon for struvite has not been described in the literature so far, although struvite is indicated as a piezoelectric material in Ref.^23^.

## Materials and methods

### Growth of struvite crystals

**Figure 1:**
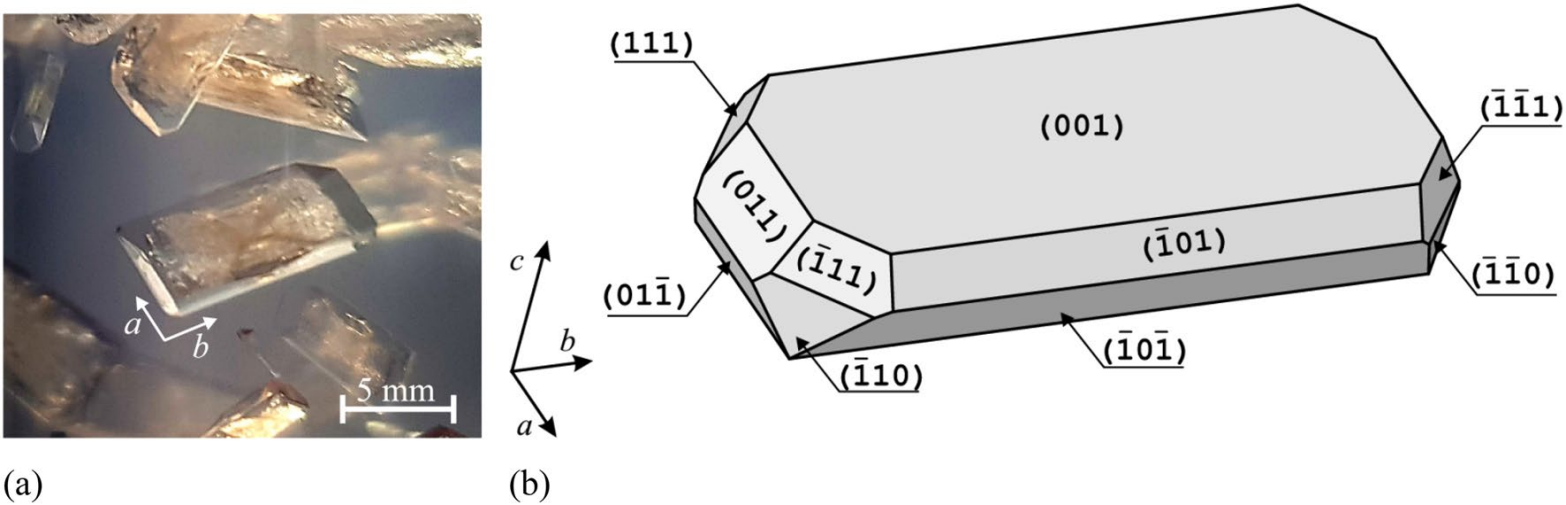
Struvite crystal (**a**) in metasilicate gel; (**b**) schematic representation of struvite habit with Miller indices of faces. Figure (**b**) reprinted from Ref. ^20^.

To measure the piezoelectric effect, it is necessary to have a crystal of a specific size in the form of a flat plate. We have grown such crystals in a metasilicate gel environment, using a single diffusion gel growth technique. We used the same crystal growth method for studies on struvite ferroelectricity. This method is described in Ref.^20^. Here, it can only be said briefly that all reagent-grade purity chemicals used were purchased from Sigma Aldrich. For the gel preparation we used anhydrous sodium metasilicate (Na_2_SiO_3_; SMS), ammonium dihydrogen phosphate (NH_4_H_2_PO_4_; ADP), and magnesium acetate tetrahydrate (Mg(CH_3_COO)_2_∙4H_2_O). The chemicals were dissolved in distilled water. A 0.5 M aqueous ADP solution and 1.07 specific gravity SMS solution were mixed in appropriate amounts to obtain a pH of 7.0. The mixture prepared in this way was poured into 19 cm long tubes with a diameter of 3 cm and allowed to gel for 24 h. After gelation, 25 ml of 1 M magnesium acetate tetrahydrate was gently poured onto the surface of the newly formed gel in test tubes and closed with a lid. The crystal growth usually lasted for three to four weeks. After this time, struvite crystals had the form of rectangular platelets about 1 cm long along the *b* axis. An example photo of such a crystal is shown in Fig. 1a. The Miller indices of the crystal faces (Fig. 1b) were determined using the X-ray diffraction method, as described in Ref.^2^.

### Piezoelectric effect measurement

Two methods were used to determine the piezoelectric properties. For both of these methods, the struvite crystal was in the form of a plane parallel plate (as shown in Fig. 1a,b). However, to measure the piezoelectric effect, the struvite crystal with surface (001) (as in Fig. 1) was polished to a size of 5.125 × 1.25 × 0.3 mm with the longest edge parallel to the *b* axis. This geometry is needed for measuring the *d*_32_ coefficient (as shown for example in Ref.^24^ in Fig. 1). Silver electrodes were deposited on opposite faces (001) and $\left( {00\overline{1}} \right)$. Miller indices of faces were determined on the basis of XRD data presented in Ref.^2^. Prior to making measurements the sample was poled in d.c. electric field at 293 K. The piezoelectric properties reached a saturation state for the poling field of strength 15 kV/cm, significantly more extensive than the value of the coercive field assumed 6 kV/cm, reported by us in Ref.^20^.

The first method used was related to measurements of piezoelectric resonances, in order to calculate the *d*_32_ piezoelectric coefficient. To find the piezoelectric frequencies, the complex admittance *Y* was measured as a function of frequency *f* using Hewlett-Packard 4192A impedance analyser. The measurements of the absolute *Y* and phase angle θ were carried out with sinusoidal voltage of 1 V. Small step frequency was used to detect the admittance changes near the resonance frequencies accurately. The forced damped harmonic oscillator model was applied to calculate the complex elastic compliances, coefficient of electromechanical coupling *k*_32_, low-frequency permittivity ε_33_ and finally, the piezoelectric coefficient *d*_32_ (Fig. 3).

In the second method which used the inverse piezoelectric effect, quasi-static strain was measured for the same poled sample as that used in dynamic method. Mathematically, the strain can be represented as follows:

1 $$\eta_{i} = d_{ij} E_{j} ,$$

where $\eta_{i}$, $d_{ij}$ and $E_{j}$ stand for strain tensor, piezoelectric coefficient, and electric field, respectively. A quasi-static method based on capacitance sensor was used to determine the piezoelectric strain ${\eta}_{3}$ in crystals under the electric field applied. *η*_3_ denotes the strain of lattice along the [001] axis. Details of the measurement method can be found in Ref.^25^. Sample deformation was induced by alternating electric field *E* of frequency 160 Hz applied to the sample and transferred via a quartz rod with one end placed on the sample surface and the other one connected to the plate of capacitor sensor with the capacity of *C*_0_. The value of *C*_0_ was adjustable, to optimize resolution depending on the magnitude of strain being measured. The frequency of 160 Hz was chosen to avoid external noise and to minimize experimental error. The surface area of the end of quartz rod touching the crystal was of the order of 0.1 mm^2^. Piezoelectric deformation appeared synchronously with the applied voltage of angular frequency ω causing changes *∆C*_p_ of sensor capacity *C*_0_. The changes in capacity *C* over time *t* can be expressed by the equation: *C*(*t*) = *C*_0_ + Δ*C*_p_ · sin(ω*t*). Δ*C*_p_ was determined through recording of the current by means of a lock-in amplifier and the piezoelectric strain amplitude ${\eta}_{3}$ was calculated from the relation

2 $$\eta_{3} = \frac{{\varepsilon_{0} S}}{d} \cdot \frac{{\Delta C_{{\text{p}}} }}{{C_{0}^{2} }},$$

where *S* is the surface of sensor capacitance plates, *d* is the thickness of the sample, and *ε*_0_ is the vacuum permittivity. From the *η*_*3*_ strain, the piezoelectric coefficient *d*_33_ can be determined from the relation

3 $$d_{33} = \frac{{\partial \eta_{3} }}{{\partial E_{3} }}.$$

Since the strain was measured on the poled crystal (surface (001)) and an electric field *E*_3_ was applied along the direction [001], the piezoelectric coefficient *d*_33_ could be determined.

### Differential scanning calorimetry (DSC) measurements

The DSC measurements were made with DSC 200 F3 differential scanning calorimeter (NETZSCH). The measurements were performed under the flow of analytical grade 6.0 nitrogen (flow rate 20.0 cm^3^/min) in the temperature range of 293.16–413.16 K with heating rate of 0.4 and 1.2 K/min, and 293.16–433.16 K with heating rate of 2.0 K/min. The samples were sealed in crucibles with internal size fitting the size of single crystals used for measurement. Each measurement was done for one single crystal. Aluminum crucibles were used with a mass difference between them smaller than 0.005 mg. Specific heat capacity of all crucibles was tested before measurements and the difference between the reference crucible and crucible containing sample was not larger than 0.01 J/g. The temperature and enthalpy were calibrated with the use of six spectrally pure standards: adamantane, In, Sn, Bi, Zn, and CsCl. Each measurement was done in triplicate (for the same heating rate and temperature range) and the mass of samples was in the range of 3.861–17.040 mg. For all repetitions of measurements for the same conditions, the calculated enthalpies were equal within reported significant figures.

### Thermogravimetry/differential thermal analysis (TG/DTA)

Thermal analysis was carried out in STA 449 F1 TG–DTA/FTIR/QMS (NETZSCH) system of thermogravimetric analyser, coupled with infrared (IR) and mass spectrometer. The samples (mass: 10.031–10.976 mg) were heated in open corundum crucibles up to the temperatures in the range of 293.16–413.16 K with heating rate of 0.4 and 1.2 K/min in analytical grade 6.0 nitrogen. The volatile products of the decomposition process were determined from simultaneously registered IR and mass spectra.

### X-ray powder diffraction

The X-ray powder diffraction (XRPD) patterns were measured in reflection mode on XPert PRO X-ray powder diffraction system equipped with Bragg–Brentano PW 3050/65 high resolution goniometer and PW 3011/20 proportional point detector. The Cu *Kα*_1_ radiation was used. The patterns were measured at 291.0(2) K in the range of 5–90° with the narrowest beam attenuator. Diamond powder was used as an internal reference. The samples were sprinkled onto the sample holders using a small sieve, to avoid preferred orientation. The thicknesses of the samples were no more than 0.1 mm. During the measurements, each specimen was spun in the specimen plane to improve particle statistics. Continuous coupled ω-2θ scan mode was used during measurement. X’Pert HighScore Plus^26^ software was used for data collection and data processing. The reference pattern was taken from Powder Diffraction File^27^.

## Results and discussion

**Figure 2:**
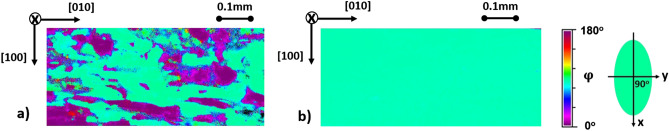
(**a**) Randomly oriented domains before poling in d.c. field. The structure of these domains is analogous to that presented in Ref. ^20^. (**b**) Single-domain state after poling in d.c. field represented by homogenous orientation of the optical indicatrix in the crystal. This homogenous orientation, observed after removing electrodes from the surfaces of poled crystal, indicates the permanent state of polarization after all piezoelectric measurements utilizing resonance method (Fig. 3).

**Figure 3:**
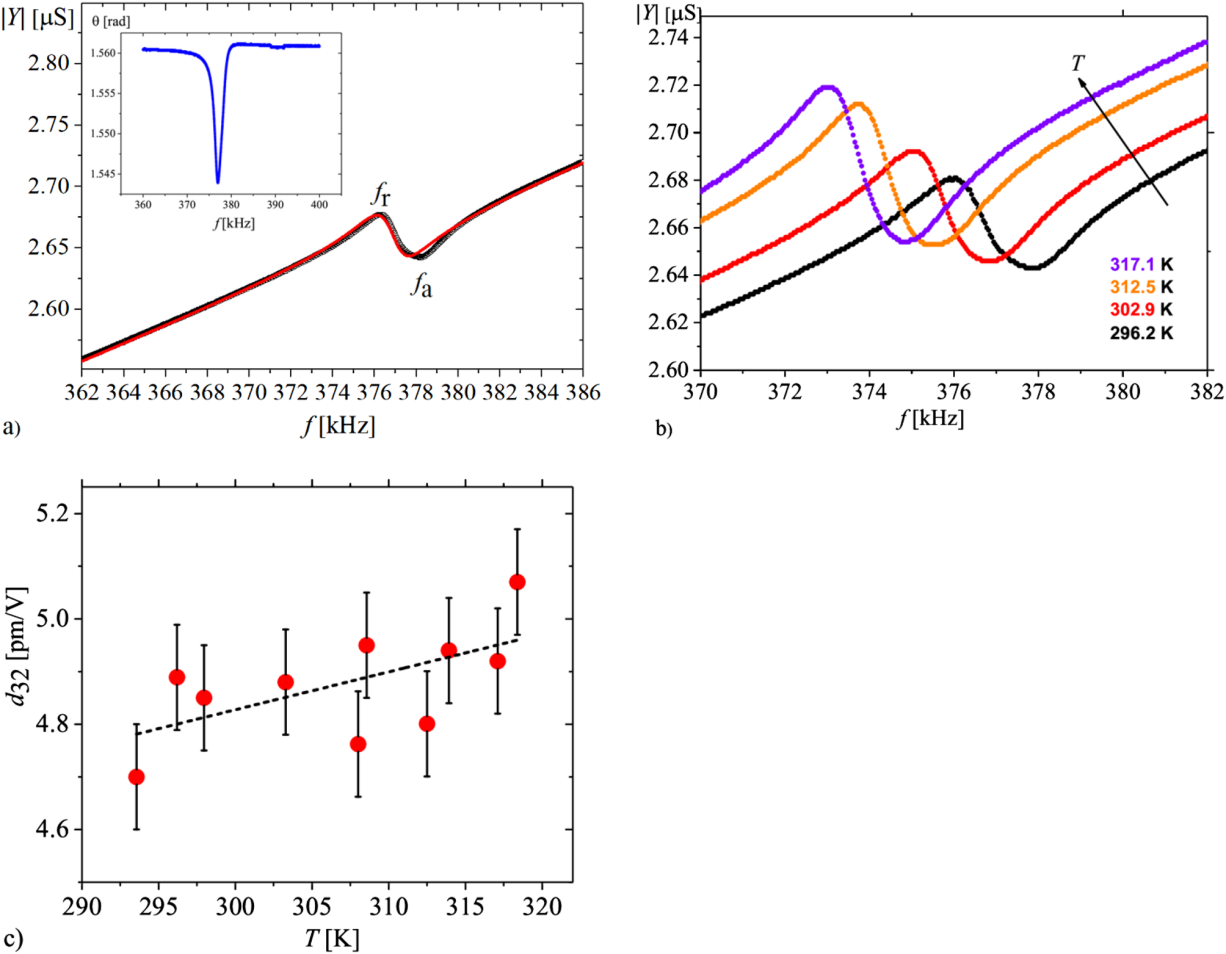
(**a**) Piezoelectric signal at 293 K, with characteristic dependence of admittance |*Y*| near piezoelectric resonance *f*_r_ and antiresonance *f*_a_. The red line represents fit to experimental data based on the model described in Ref. ^30^. The inset represents changes of phase shift θ in the relationship *Y* = |*Y*| e ^iθ^. (**b**) The temperature evolution of piezoelectric resonances shows a shift of resonances towards lower frequencies with increasing temperature. All dependencies in Figs. a and b have been obtained for the strength of the measuring field amounting to 0.035 V/cm. *c*) *d*_32_ piezoelectric coefficient in a function of temperature in low temperature range. Standard deviation for each measuring point is shown. The dashed black line is the linear fit to the measurement points showing a weak increase in *d*_32_ with temperature.

For the sample prepared according to the procedure described in Sect. Piezoelectric effect measurement, the *d*_32_ piezoelectric coefficient was measured using the resonance method. Prior to measurements, the sample was polished to the thickness of 0.3 mm. Struvite has a good (001) cleavage plane^28,29^ perpendicular to the polar axis which greatly facilitates the preparation of thin plates. Figure 2a presents randomly oriented domains for the crystal before poling process, while Fig. 2b presents single-domain state of the crystal after poling in the d.c field. To visualise the domains, we have used the technique described in Ref.^20^. After poling, according to procedure mentioned in Sect. Piezoelectric effect measurement, the *Y*(*f*) dependencies were collected as a function of temperature. These dependencies are presented in Fig. 3. With temperature increase the resonance frequency shifted towards lower frequencies. Based on these data the temperature dependence of the *d*_32_ piezoelectric coefficient, estimated from the resonance frequency *f*_r_ (local maximum od admittance, *Y*) and antiresonance frequency *f*_a_ (local minimum of admittance, *Y*), was drawn. The method for determination of piezoelectric coefficients is described elsewhere^30^.

**Figure 4:**
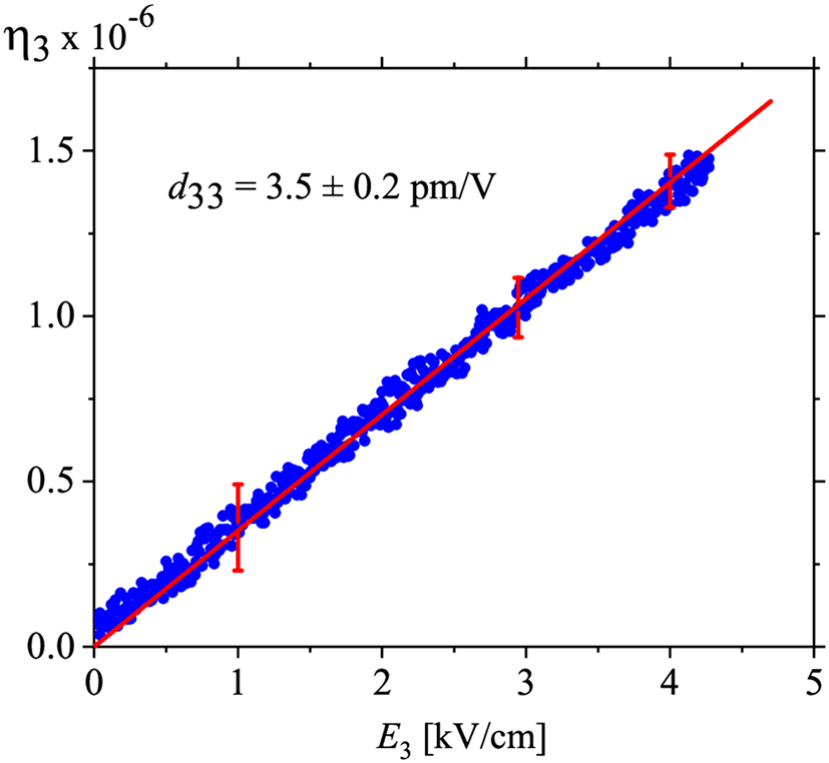
Strain *η*_3_ as a function of a.c. electric field strength *E*_3_ of frequency of 160 Hz at 300 K. To keep the crystal in thermodynamic equilibrium, slowly and continuous change of the *E*_3_ field with the rate of 0.075 kV/minute was applied. Since the electric field was applied in the direction [001], we have measured the *d*_33_ piezoelectric coefficient. The red straight line represents a fit to the relationship describing the piezoelectric effect *η*_3_ = *d*_33_ *E*_3_.

**Figure 5:**
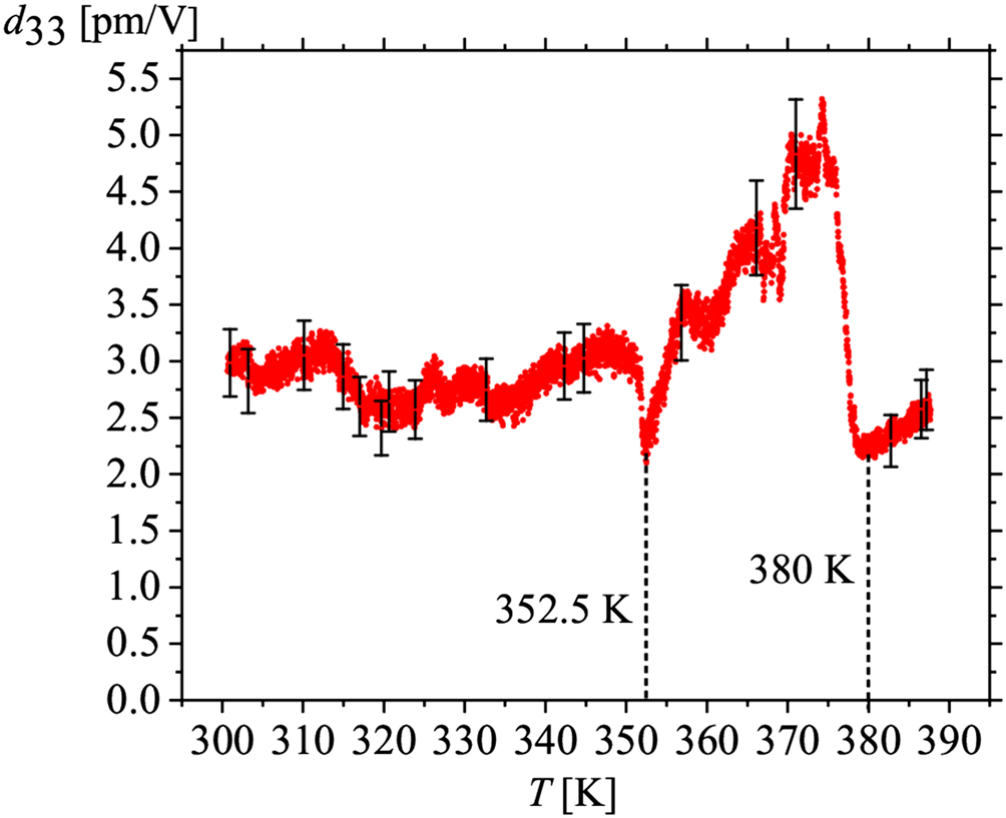
Temperature dependence of *d*_33_ coefficient measured for the a.c. electric field strength of 4 kV/cm and the frequency of 160 Hz. Experiment has been performed for the same crystal for which data at 300 K are presented in Fig. 4. Above 380 K the crystal is already of dittmarite structure (see further in the text). Since dittmarite structure is of orthorhombic symmetry, the piezoelectric effect was also observed. However, in this experiment we could not establish the crystallographic orientation of the crystal after being transformed to dittmarite at 380 K. Thus, above this temperature, the piezoelectric tensor might not be ascribed as *d*_33_.

We have found that the piezoelectric properties described by *d*_32_ are not strongly dependent on temperature in the range from room temperature to 320 K. We have repeated the dependence as that in Fig. 3 a few times and no changes have been found. Since we wanted to measure the *d*_33_(*T*) dependence (Figs. 4 and 5) for the same poled crystal, we did not continue the measurements by resonance technique to a higher temperature to avoid the crystal decomposition.

Figure 4 shows the linear dependence of strain *η*_3_ versus electric field *E*_3_ in the range of the strengths up to 4 kV/cm. We have chosen this field strength in order not to destroy the single domain state (the coercive field for struvite, as reported in Ref.^20^ is of the order of 6 kV/cm). It was confirmed by a linear function of the polarization *P* versus *E* observed simultaneously throughout the quasi-static measurements. The *d*_33_ piezoelectric module has been calculated from the linear *η*_3_(*E*_3_) run (Eq. (3)) and amounts to 3.5 ± 0.2 pm/V.

However, essential changes have been found in a much broader temperature range. Namely, the piezoelectric properties are observed up to 390 K (Fig. 5). Up to the temperature of 352.5 K, the value of the *d*_33_ piezoelectric coefficient oscillates around the value of 3.5 pm/V, which is characteristic for the temperature of 293 K. As can be seen in Fig. 5, above 352.5 K, the *d*_33_ piezoelectric coefficient increases irregularly and finally the dependence *d*_33_(*T*) drastically goes to a minimal value for 380 K. Interestingly, above this temperature, we still observe the piezoelectric response. This sudden decrease in the value of the *d*_33_ coefficient may indicate that at 380 K we are dealing with the transformation of struvite into a different structure. The irregularity that appears above the temperature of 352.5 K may indicate that this transformation begins just from this temperature. It should also be kept in mind that the results presented in the Fig. 5 were obtained for a temperature rate equal to 0.4 K/min.

**Figure 6:**
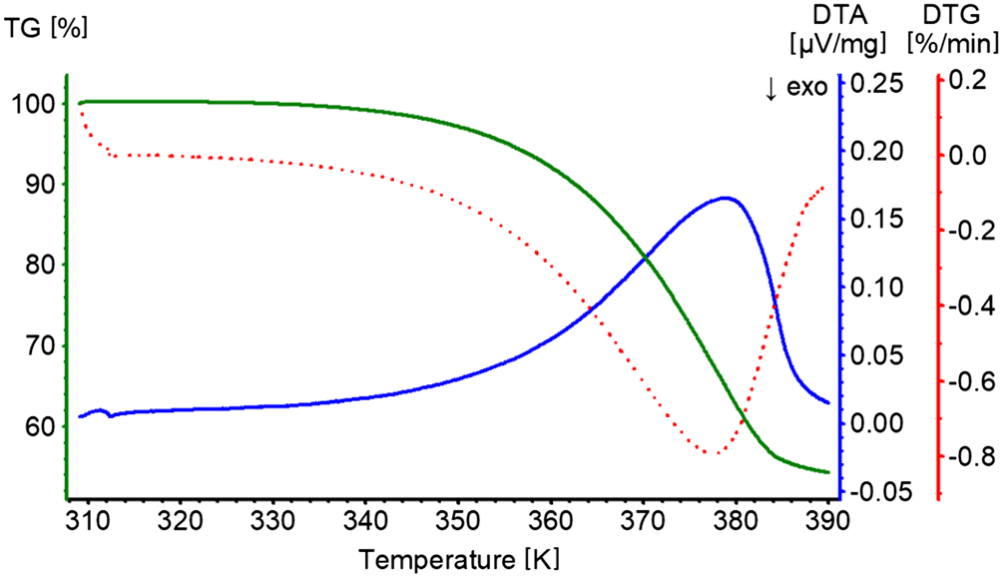
TG, DTA, and DTG (derivative thermogravimetric) curves of struvite heated with a heating rate of 0.4 K/min in an open crucible.

**Figure 7:**
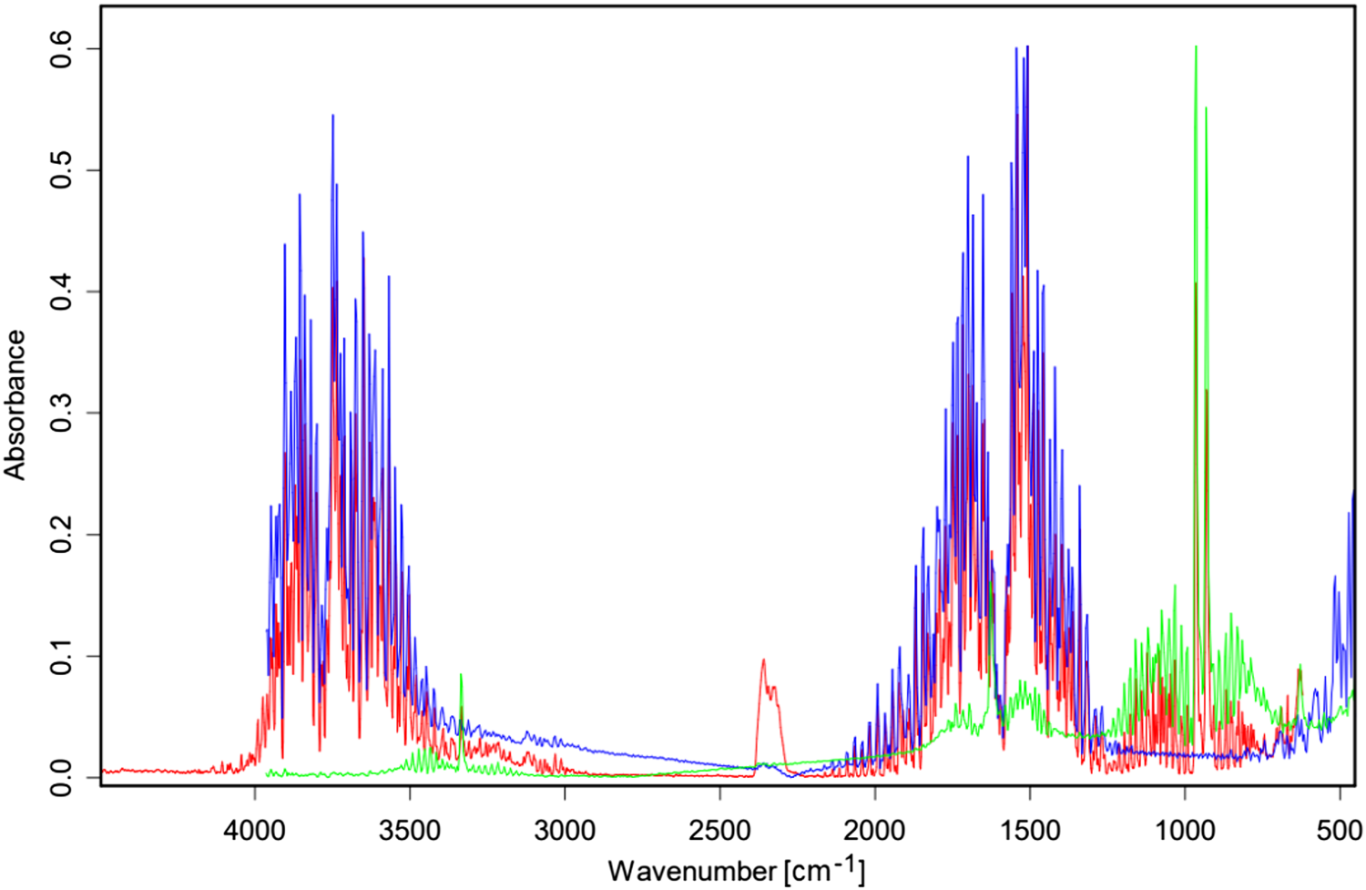
Fourier Transform Infrared (FTIR) spectrum of gaseous products formed during heating of struvite in an open crucible (registered at 340 K)-red, water reference spectrum-blue, ammonia reference spectrum-green.

To test whether struvite undergoes any transformation with increasing temperature, struvite was heated under controlled conditions. Initially, struvite was heated in open crucibles. Such heating leads to its decomposition to magnesium hydrogen phosphate (MgHPO_4_). The process of decomposition starts after reaching the temperature of 333 K and from the beginning it is accompanied by releasing gaseous water and ammonia molecules (Figs. 6 and 7). Mass spectra contain two principal signals with m/z of 17 and 18 (m/z is the mass-to-charge ratio value), which also confirms the formation of NH_3_ and H_2_O as gaseous products. This finding is in agreement with the previously published results concerning the thermal decomposition of struvite^31^. Despite the accordance with the literature, this measurement did not answer the question of what happens to struvite at 380 K, as it is observed in the measurement of piezoelectric properties. That is why we have decided to measure the thermal decomposition of struvite under conditions as close as possible to those during the piezo-effect measurements. To get the piezoelectric response, silver electrodes were applied to faces (001) and $\left( {00\overline{1}} \right)$. Assuming that these electrodes could influence the process of thermal decomposition of struvite, we measured the process of thermal decomposition by placing struvite in a sealed crucible matching the crystal size (see Materials and Methods). The crystals adhered to the bottom and cover of the crucible with faces (001) and $\left( {00\overline{1}} \right)$, respectively. This procedure of the crystal faces adhering to the crucible was to imitate the existence of silver electrodes.

**Figure 8:**
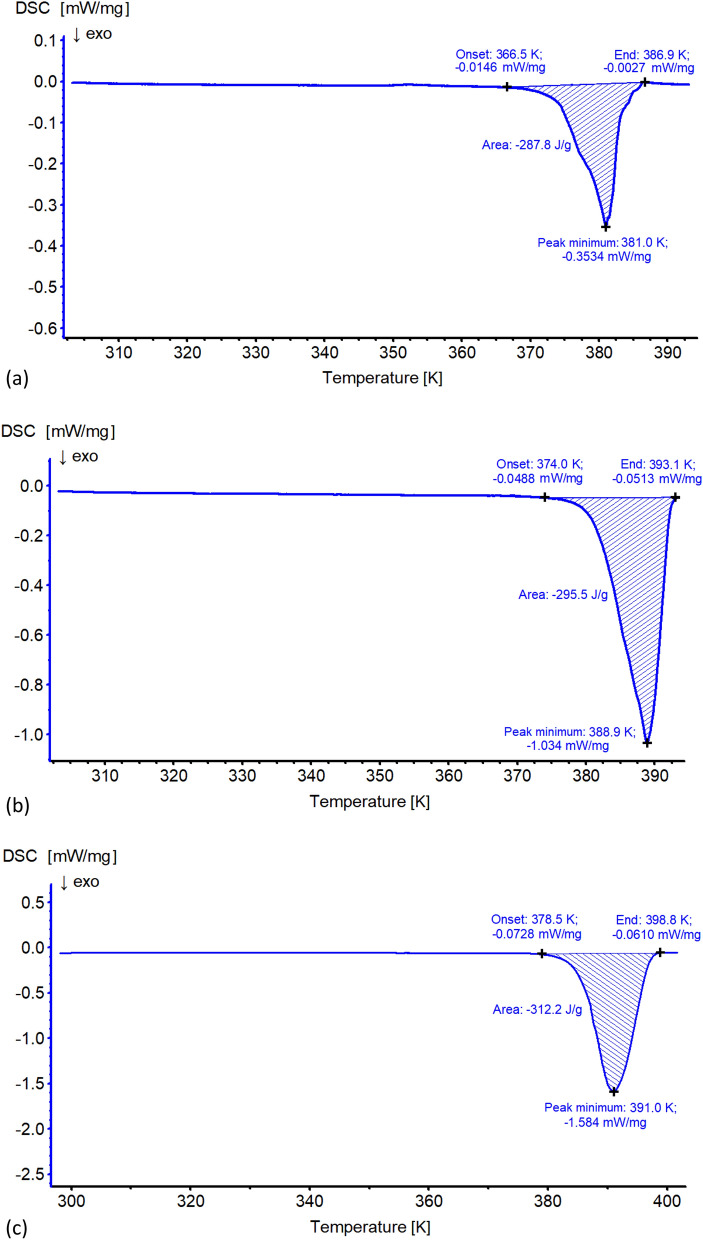
The DSC curves for struvite for different heating rates: (**a**) 0.4 K/min, (**b**) 1.2 K/min, (**c**) 2.0 K/min.

Changes﻿ in the environment of the crystal influence both the decomposition process and the resulting product. We have checked that single crystals of struvite heated in sealed crucibles are stable in chemical composition up to 366.5–378.5 K, depending on the heating rate (Fig. 8, Table 1).

**Table 1:** DSC results for struvite → dittmarite conversion process.

Heating rate [K/min]	*T*_s_ [K]	*T*_max_ [K]	*T*_t_ [K]	*H* [J/g]
0.4	366.5	381.0	386.9	− 287.8
1.2	374.0	388.9	393.1	− 295.5
2.0	378.5	391.0	398.8	− 312.2

*T*_s_ temperature of process start, *T*_max_ temperature at maximum of energetic effect, *T*_t_ temperature of process termination, *H* specific enthalpy of the process.

Then, for all samples, a well distinguishable exothermic process is observed which leads to development of a thermally stable product. In other words, under experimental conditions, struvite is transformed into another stable product. The temperature for completion of this transformation varies from 386.9 to 398.8 K depending on the heating rate (Fig. 8 and Table 1). The typical effect of an increase in decomposition temperature and measured specific enthalpy with increase in heating rate is observed (Table 1), resulting from differences in energy flux for experiments with different set up.

**Figure 9:**
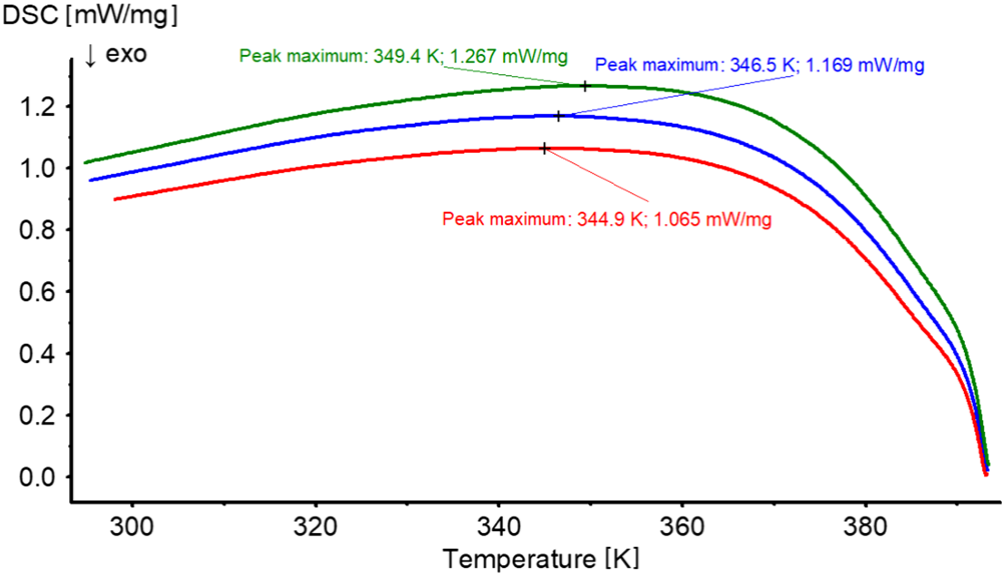
The DSC curves after struvite phase transformation for different cooling rates: 0.4 K/min (red), 1.2 K/min (blue), 2.0 K/min (green).

To make sure that the final product obtained on heating is stable, we performed another experiment. After phase transformation, the samples were cooled to the initial temperature. As can be seen in Fig. 9, cooling the samples back to the starting temperature did not produce any sharp or evident energetic effect. Only a smooth change of enthalpy is observed in the whole measurement range, and this proves that the process observed on heating is irreversible and the final product is stable. There are reports in the literature (e.g. Ref.^32^) that dittmarite can turn into struvite in a sufficiently long time when exposed to high humidity air. We did not observe such a process under the conditions of our experiment.

For the heating rate of 0.4 K/min, in the case of piezo-effect measurements, we obtained an irregular increase in the value of *d*_33,_ starting from the temperature of 352.5 K (Fig. 5). These irregularities may indicate a change in the chemical composition of the test sample. Comparing this result with the result obtained from DSC measurements for the same heating rate, we see that struvite is stable in terms of its chemical composition up to the temperature of 366.5 K (Fig. 8a). At first glance, it may seem that these temperatures do not coincide. However, it should be borne in mind that despite the fact that during the DSC measurements we tried to imitate the conditions prevailing during the piezo-effect measurement as well as possible, these conditions were not identical. This is how we explain the difference in these temperatures. From the measurements of the piezoelectric effect (Fig. 5) we can also see that at the temperature of 380 K we observe a sharp decrease in *d*_33_, but above this temperature we still observe a piezoelectric response. As suggested earlier, this may be related to the decomposition of struvite into a different structure. In the DSC tests, at the same heating rate of 0.4 K/min, the conversion of struvite into a different structure finalizes at 386.9 K (Fig. 8a and Table 1). Because the piezoelectric and DSC experiments were not performed at exactly the same conditions, these two temperatures coincide quite well.

**Figure 10:**
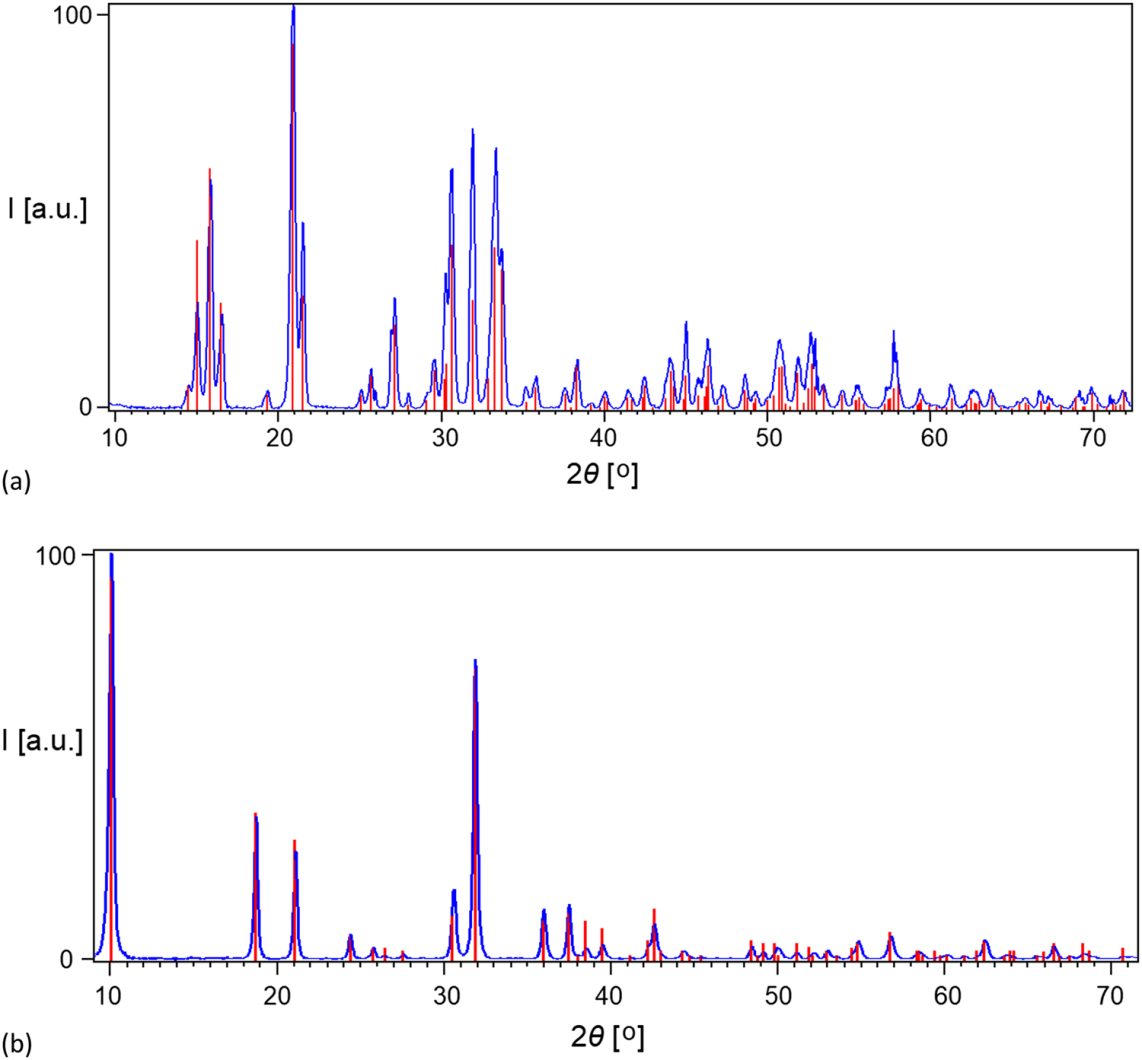
The XRPD pattern of struvite (**a**) before heating (blue), and struvite reference pattern (red) with PDF-2 code 01-071-2089, and (**b**) struvite heated (blue) in sealed crucible up to 393 K and dittmarite reference pattern (red) with PDF-2 code 00-036-1491.

So, the question remained concerning the structure and chemical composition of this end product. To check that, XRPD patterns were registered. In all cases, dittmarite is the sole phase observed (Fig. 10). Dittmarite is a mineral with the chemical formula of MgNH_4_PO_4_·H_2_O (chemical formula for struvite is MgNH_4_PO_4_⋅6H_2_O). It crystallizes in the orthorhombic system with the P*mn*2_1_ space group, i.e. the space group is the same as in the case of struvite. The unit cell parameters of the obtained dittmarite (determined on the basis of the XRPD pattern, Fig. 10b) are as follows: *a* = 5.620 Å, *b* = 8.745 Å, *c* = 4.783 Å. This means that when struvite is heated under the conditions described, it transforms from magnesium ammonium phosphate hexahydrate into magnesium ammonium phosphate monohydrate, with a simultaneous change in unit cell parameters.

To evaluate the influence of higher heating rates, as well as prolonged annealing time, on the final product, the single crystal put in the sealed crucible was inserted into the furnace, heated to 393 K and kept there for 90 min. In such experiment, dittmarite was also observed as the final product. This proves that the formation of dittmarite in sealed crucible is independent of the heating rate and annealing time. All measured dittmarite samples had a high degree of crystallinity (amorphous phase scattering was not observed in any XRPD pattern) and had a large size of crystallites (the observed broadening of reflections was the same as for microcrystalline standard and originated only from instrumental broadening). It should be noted that in all crucibles unsealed after DSC measurement the crystals retained their shape. The transformation of struvite into dittmarite has been reported many times in the literature^32–37^. The researchers indicated that such a transformation in air (not in solution) depends on temperature and heating rate, which is also confirmed by our research.

**Figure 11:**
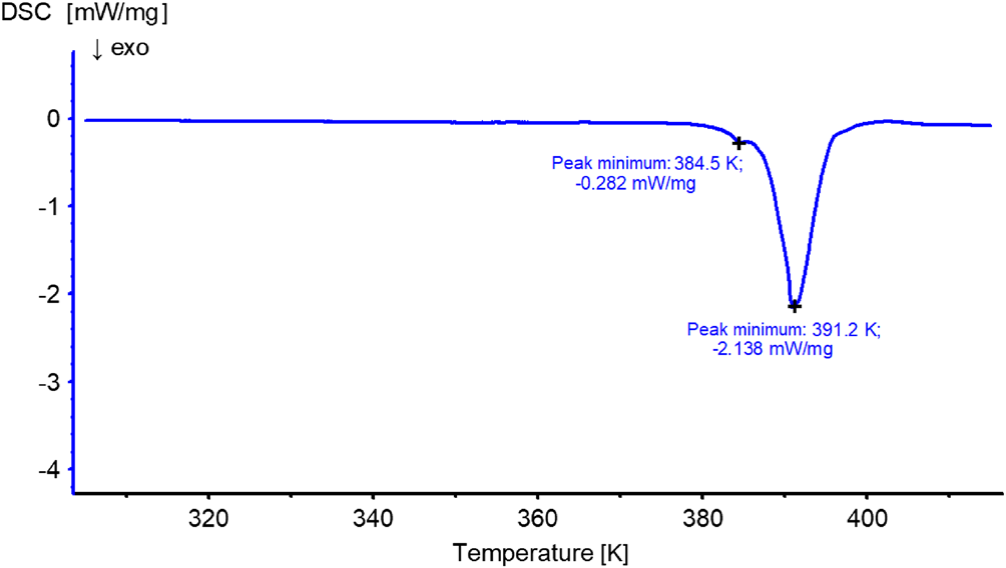
The DSC curves of struvite crystal possessing line defect. The peak at 384.5 K is associated with the existence of a dislocation.

It must be outlined that imperfections of struvite crystals alter the smoothness of the DSC curve and cause the occurrence of additional local energy maximum. For example, the presence of visible dislocation in crystal led to the emergence of additional maxima at 384.5 K for temperature rate of 2.0 K/min (Fig. 11) and breakage of crystal into two parts, along observed dislocation. This can be explained by strain existing in the crystal after water has been released which splits the crystal exactly in the defect area, i.e. in the weakest point of the crystal lattice. In all the measured crystals with defects, the local maximum emerges from the global maximum when water loss is sufficient for strain relaxations, and it occurs before the global maximum of the struvite → dittmarite conversion process.

The observed results imply that the complete closing of the single crystal of struvite in a crucible (or any other vessel) is sufficient for crystalline dittmarite to be formed when heating above 370 K.

The FTIR, DSC and XPRD tests clearly show that struvite heated in sealed crucibles remains stable in terms of chemical composition up to the temperature of 366.5–378.5 K, depending on the heating rate. For the heating rate of 0.4 K/min, for which piezo-effect measurements were made, the temperature of 352.5 K for which irregularities in the variability of *d*_33_ are observed—which may indicate a change in the chemical composition of the sample—corresponds quite well with the results obtained from DSC tests, taking into account the fact that measurement conditions were not identical in both measurement techniques. As a consequence, the process of changing the chemical composition of struvite leads to the final product, which is dittmarite.

Based on these results, we can conclude that the piezoelectric response observed above the temperature of 380 K (Fig. 5) is a dittmarite response. Dittmarite is non-centrosymmetric, it crystallizes in the same space and point group as struvite and exhibits a piezoelectric effect either. Hence, this article presents the first experimental evidence regarding the piezo-effect in dittmarite to the best of our knowledge. However, we were unable to determine precisely the coefficient of the piezoelectric tensor.

**Figure 12:**
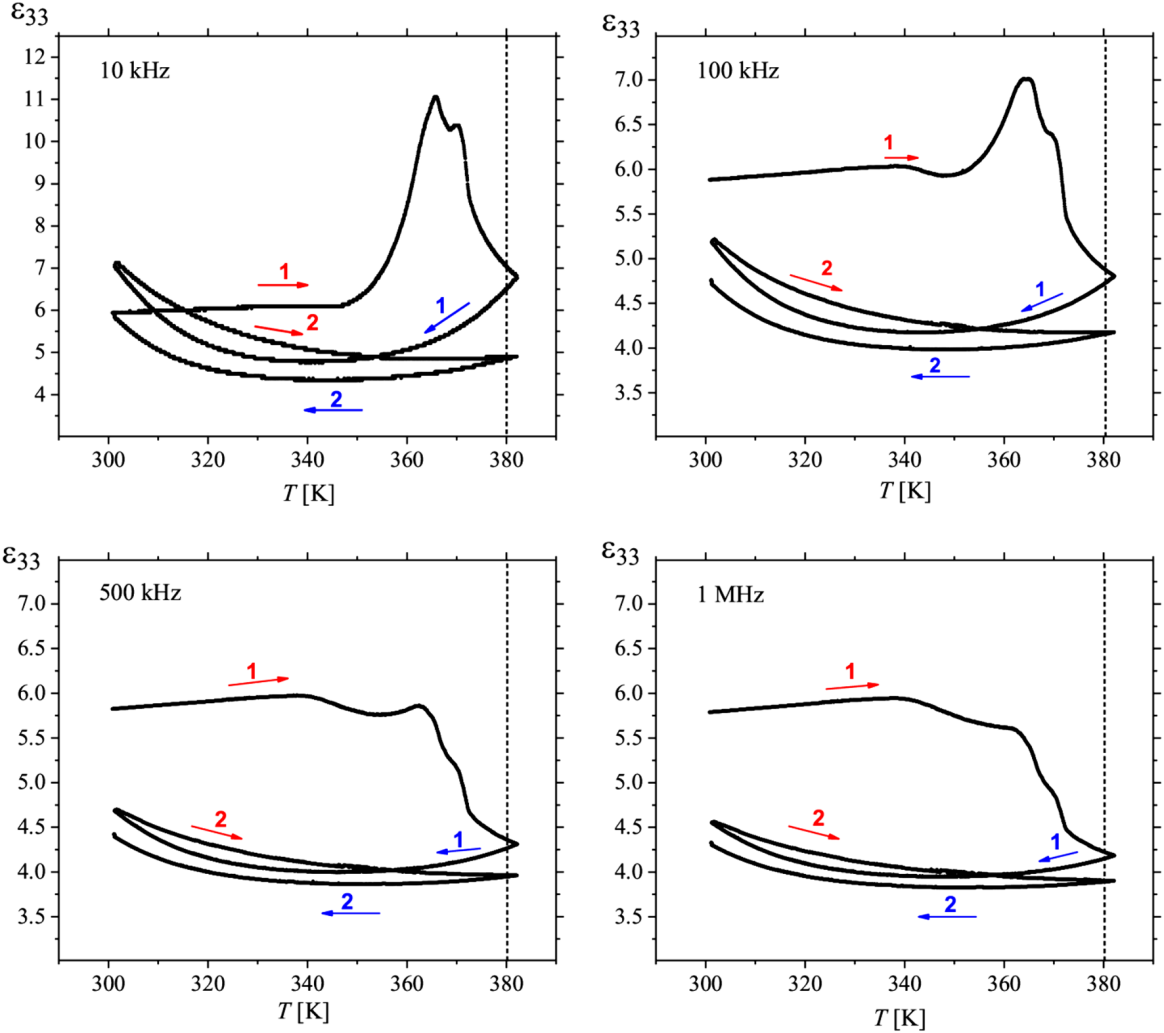
Temperature and frequency dependence of permittivity ε_33_, measured for the temperature rate of 0.4 K/min., demonstrating the transformation of struvite into dittmarite structure near 380 K. The fastest changes in all ε_33_(*T*) dependencies in the first cycle, marked in red 1, start above 350 K (for frequencies of 10 and 100 kHz) and 360 K (for frequencies of 500 kHz and 1 MHz). The permittivity ε_33_ measured at frequencies below the piezoelectric resonances (10 kHz and 100 kHz) increases before struvite-dittmarite transformation at 380 K. The same behaviour reveals temperature changes of the *d*_33_, which rises before transformation point because its value depends directly on the ε_33._ Thus, the data for low frequencies 10 kHz and 100 kHz corresponds well to temperature behaviour of the piezoelectric coefficients: *d*_33_ and *d*_32_. Red and blue arrows indicate heating and cooling processes, respectively.

The struvite-dittmarite transformation at 380 K was also confirmed through measurements of the permittivity as a function of temperature. Figure 12 shows dependencies of ε_33_(*T*), for four different frequencies, in the temperature range in which transformation to the dittmarite structure takes place.

As shown in Fig. 12, the fastest changes in the course of ε_33_(*T*) start above 350 K, which is approximately the temperature at which the change in the chemical composition of struvite begins. Moreover, this Figure shows a weak dielectric dispersion in the temperature range 300–380 K and frequency range 10 kHz to 1 MHz. The most important result is a clear transformation into dittmarite structure at 380 K. After exceeding this temperature, the dependence ε_33_(*T*) no longer resembles that for struvite and decreases almost twice. The fact that ε_33_(*T*) is not repeatable after two heating–cooling cycles means that to get complete transformation into dittmarite, one has to be at the temperature higher than 380 K. As results from DSC measurements (Fig. 8a and Table 1), the temperature of completion of struvite transformation into dittmarite is 386.9 K for the heating rate of 0.4 K/min.

As our results presented in this paper show, piezoelectric coefficients *d*_33_ and *d*_32_ observed for struvite crystals at room temperature are 3.5 pm/V and 4.7 pm/V, respectively. Struvite is a mineral, so it is worth comparing the piezo-response of other minerals. In the review^38^ piezoelectric constants *d*_33_ for various minerals are presented. One of the lowest values of *d*_33_ is exhibited by mineral boracite, for which the *d*_33_ is 0.6 pm/V at room temperature. For ice, *d*_33_ is 2 pm/V at 0 °C, and for tourmaline, *d*_33_ is 33 pm/V at room temperature. As it results from Ref.^38^, the highest value of *d*_33_ exhibits mineral stibiotantalite, for which *d*_33_ is 370 pm/V at room temperature. It must therefore be concluded that struvite has a *d*_33_ value which is typical for minerals, but within the range of smaller values. The *d*_32_ value obtained for struvite is much more difficult to compare with the values for other crystals because it is determined much less frequently. We have managed to find the *d*_32_ coefficient values for two minerals: magnetite^38^ and changbaiite (lead niobate)^39^. For magnetite, *d*_32_ and *d*_33_ values are given for − 269 °C as higher than 0.06 pm/V and 0.25 pm/V, respectively. For changbaiite, the coefficients *d*_32_ and *d*_33_ are equal to 24 pm/V and 60 pm/V^39^, respectively. Comparing the *d*_32_ and *d*_33_ values for magnetite and changbaiite may indicate that the *d*_32_ coefficients are smaller than the *d*_33_ ones. A similar relationship is often observed also for crystals, which are not minerals (e.g. Ref.^40^). In the case of struvite, we observe the inverse relationship. This means that this issue requires additional research. It is known that the physical properties of the crystal, including the piezoelectric properties, depend on the non-centrosymmetric structure of the crystal. The structure of struvite is known to consist of PO_4_^3-^ and NH_4_^+^ tetrahedra and Mg[H_2_O]_6_^2+^ octahedra bound by hydrogen bonds^41^. PO_4_ tetrahedra are regular, while Mg[H_2_O]_6_^2+^ octahedra are very distorted^41^. In addition, there are net electric dipole in struvite resulting from the existence of spontaneous polarization^20^. It is known that piezoelectric properties can be influenced by symmetry elements resulting from the space group, but in the case of struvite, the contribution of these elements to piezoelectric properties should be analysed from the point of view of distorted octahedra and net moment dipole. Only such an analysis can provide credible evidence for the origin of piezoelectricity in struvite as well as explain the relationship between *d*_32_ and *d*_33_. This remains a challenge for future research.

## Conclusions

To the best of our knowledge, this paper presents the first experimentally confirmed piezoelectric properties of struvite. We have found that at room temperature the *d*_33_ coefficient is 3.5 pm/V, while that of *d*_32_ is 4.7 pm/V. These are typical values for minerals, within the range of lower values. These coefficients do not change significantly with increasing temperature. However, struvite reveals the piezoelectric properties up to a specific temperature that depends on the rate at which the crystal is heated. At the rate 0.4 K/min, struvite shows stable piezoelectric properties up to 352.5 K. Above this temperature, for that heating rate, struvite begins to lose ammonia and crystallization water and undergoes transformation into a monohydrate form, i.e. dittmarite. The temperature of this transformation is dependent on heating rate of the crystal. The higher the heating rate, the higher the temperature of this transformation. Dittmarite is stable and does not convert to struvite during the cooling process. Dittmarite, like struvite, is non-centrosymmetric and also exhibits piezoelectric properties that, to the best of our knowledge, have not been reported yet.

There are still many challenges to be faced regarding the crystallization of struvite, either in wastewater treatment plants or in the context of infectious urinary stones. We believe that studies concerning the piezoelectric effect reported in this paper will introduce further research into other struvite-related phenomena.
